# Interactive effects of calcium-nitrogen combined stress and nitrogen forms on nitrogen metabolism and physiological ecological response mechanisms of *Toona sinensis* Seedlings

**DOI:** 10.3389/fpls.2026.1806019

**Published:** 2026-04-10

**Authors:** Hongtao Hang, Guoling Guo, Chuanting Li, Yunfei Zhang

**Affiliations:** 1 School of Karst Science, Guizhou Normal University, Guiyang, China; 2 State Engineering Technology Institute for Karst Desertification Control, Guiyang, China; 3 State Key Laboratory of Environmental Geochemistry, Institute of Geochemistry, Chinese Academy of Sciences, Guiyang, China

**Keywords:** calcium stress, karst, nitrogen form, nitrogen level, physiological response, *Toona sinensis*

## Abstract

Karst habitats are characterized by composite stress conditions featuring high calcium, low nitrogen, and a unique nitrogen form profile ("low ammonium, high nitrate"), which profoundly shape plant physiological adaptation strategies. Current research often examines the isolated effects of either calcium or nitrogen, failing to adequately explain their interactions in karst environments. Therefore, investigating the interactive effects of nitrogen level and form under high calcium stress on the growth and physiological traits of *Toona sinensis* seedlings is crucial for understanding plant adaptation mechanisms in this region. A hydroponic experiment was conducted to investigate the interactive effects of nitrogen level and form on the growth and physiological characteristics of *Toona sinens* is seedlings under high calcium stress. Two calcium levels (5 mM and 120 mM), three nitrogen levels (3.75, 7.5, and 15 mM), and three nitrate-to-ammonium ratios (0:100, 50:50, and 100:0) were applied. Morphological, physio-biochemical, and mineral element indices were measured. The results showed that under normal calcium conditions, the mixed nitrogen form (50:50) was optimal at medium and high nitrogen levels. However, when the calcium concentration was increased to 120 mM, high-calcium stress completely overrode physiological responses and disrupted core metabolic functions, leading to a collapse of physiological functions in the seedlings. Specifically, a burst of reactive oxygen species (ROS) exacerbated membrane lipid peroxidation, with root malondialdehyde (MDA) content surging to 7.68 times that observed under normal calcium conditions. The antioxidant system (SOD, POD) became dysfunctional, and the activities of nitrogen-metabolizing enzymes (NR, GS) were almost completely inhibited, thereby hindering the conversion of inorganic nitrogen to organic nitrogen. Simultaneously, mineral ion homeostasis (e.g., Ca/Mg ratio) was severely disturbed, resulting in the breakdown of metabolic homeostasis. These findings indicate that *T. sinensis* can achieve adaptive growth under normal calcium conditions by adjusting nitrogen utilization strategies, whereas irreversible physiological collapse occurs under high calcium stress. High calcium acts as a limiting factor by disrupting redox balance, nitrogen metabolism, and ion homeostasis. In karst regions, a nitrate-to-ammonium ratio of 1:1 is recommended for fertilization under normal soil calcium conditions; however, in high-calcium habitats, priority should be given to reducing calcium levels rather than adjusting nitrogen levels or forms.

## 1 Introduction

Karst rocky desertification represents a critical constraint on regional ecological restoration and land productivity. The soil environment in these areas exhibits extreme characteristics of high calcium, low nutrient availability, and ecological fragility (Huang et al., 2007; Sheng et al., 2015). *T.sinensis*, a native tree species valued for both its ecological role in soil and water conservation and its economic potential, is considered a pioneer species for the remediation of rocky desertification (Wang et al., 2008; Chen, 2006). However, the early establishment and survival of *T.sinensis* seedlings in karst regions are often challenged by the dual stress of high calcium and low nitrogen availability with an atypical nitrogen form profile. Research indicates that soil calcium content in the karst areas of Southwest China can be 2–3 times higher than in non-karst regions, while nitrogen is generally deficient and predominantly present in the nitrate form, with ammonium being relatively low (Cao et al., 2003; Liu et al., 2004; Zhu et al., 2016). This coupled imbalance of calcium and nitrogen likely constitutes a key bottleneck limiting the successful colonization of *T.sinensis* seedlings.

Calcium, as an essential second messenger and structural component in plants, is widely involved in processes such as cell wall formation, membrane stability maintenance, ion balance, and signal transduction. However, its biological effects exhibit a typical dose-dependent pattern. Optimal calcium concentrations can enhance a plant’s resistance to environmental stress; for instance, exogenous Ca²^+^ can improve salt-alkali tolerance by modulating the antioxidant enzyme system and Ca²^+^-ROS signaling crosstalk (Hao et al., 2024). Conversely, excessive calcium inhibits plant growth through dual pathways of nutrient element antagonism and disruption of cellular signaling. At the nutritional level, high calcium strongly interferes with the uptake and transport of elements such as Mg, K, P, and Fe, thereby affecting photosynthesis and enzymatic metabolism (Palani and Indirani, 2019; Rao et al., 2025). At the signaling level, disruption of cytosolic Ca²^+^ homeostasis can trigger abnormal signaling cascades, leading to dysregulated metabolic programming (Rengel and Zhang, 2003; Borghesi et al., 2013; Ren et al., 2023). Previous studies have shown that under high calcium stress, plants often exhibit phenotypes such as growth inhibition, chlorophyll degradation, and aggravated oxidative damage (Peng et al., 2020), reflecting the physiological progression from ion toxicity to oxidative stress.

Nitrogen is a key element constituting biomacromolecules and photosynthetic apparatus, with its availability and form profoundly influencing plant growth. The effects of nitrogen typically follow a pattern of inhibitory at low levels, promotive at optimal levels, and toxic at excessive levels. Low nitrogen availability limits biomass accumulation and chlorophyll synthesis (Gao, 2021), whereas an optimal nitrogen supply enhances photosynthetic nitrogen assimilation and promotes growth (Zhao L. et al., 2024). Conversely, nitrogen excess can inhibit photosynthetic machinery, disrupt nitrogen metabolic flux, and induce oxidative stress. Additionally, nitrogen form serves as another critical regulatory factor. Numerous studies have shown that, compared to sole ammonium or nitrate nutrition, an appropriate mixed nitrate-ammonium supply (e.g., 50:50) often achieves the best balance in growth, photosynthetic performance, and nitrogen use efficiency across various crops (Song et al., 2021). This suggests that mixed nitrogen sources generally offer advantages in coordinating carbon and nitrogen metabolism and maintaining pH homeostasis. However, their synergistic or antagonistic mechanisms under stress conditions such as calcium stress remain poorly understood.

A foundation of research exists regarding the calcium and nitrogen nutrition of *T.sinensis*. Concerning calcium stress, its response exhibits a threshold, with excessive calcium inhibiting seed germination and seedling growth (Liu et al., 2016, 2021). In terms of nitrogen nutrition, both deficiency and excess disrupt the activity of key nitrogen metabolism enzymes and secondary metabolic pathways (Qiao et al., 2016; Zhao H. et al., 2024; Yu et al., 2024). Studies on nitrogen forms indicate that the nitrate-to-ammonium ratio significantly affects the growth of *T.sinensis*, with excessive nitrate showing a pronounced inhibitory effect (Shi et al.2025; Yu et al., 2024). However, the interactive effects of calcium-nitrogen combined stress, particularly how high calcium levels alter the utilization strategies of plants in response to nitrogen forms, remain poorly understood. In this study, 120 mM was set as the high-calcium stress level based on systematic results from preliminary seed germination experiments of *T.sinensis* and comprehensive consideration of calcium tolerance at the seedling stage. In the preliminary experiment, three calcium sources, namely CaCl_2_, Ca(NO_3_)_2_, and Ca(HCO_3_)_2_, were used with 14 concentration gradients ranging from 0 to 200 mM. The results showed that the germination percentage and radicle length were significantly decreased by 73.34% and 98.91%, respectively, at 100 mM under CaCl_2_ treatment. At 120 mM Ca(NO_3_)_2_, the germination percentage was markedly reduced by 58.23% compared with the control (P < 0.05).At 160 mM Ca(HCO_3_)_2_, the germination percentage was significantly decreased by 60% compared with the control (P < 0.05).Considering that seedlings usually exhibit a higher calcium tolerance threshold than seeds during germination due to their more developed root absorption system and stronger ion compartmentalization ability, and that Ca(NO_3_)_2_ was applied in the hydroponic system of this study to ensure comparability with the preliminary experiment, 120 mM was selected as the high-calcium stress level for seedlings.

## 2 Materials and methods

### 2.1 Plant material

Plump and disease-free seeds of *T.sinensis* were selected for the experiment. Following the Rules for Forest Tree Seed Testing (GB/T 2772-1999, 1999), the seeds were soaked in distilled water at 50 °C for 24 hours. They were then sown in a 12-cell seedling tray filled with perlite for germination and initial seedling development. The seedlings were grown in a controlled environment with a 12 h/12 h photoperiod, a constant temperature of 25 °C, and appropriate light intensity. During this stage, they were irrigated with half-strength Hoagland nutrient solution using a hydroponic system. When the seedlings had developed 2–3 true leaves and exhibited uniform growth, healthy individuals were selected as experimental material.

### 2.2 Experimental design

A completely randomized block design was employed for the hydroponic experiment. The study included two calcium (Ca) levels: 5 mM (normal Ca) and 120 mM (high CA, The 120 mM high-calcium level was determined based on the critical threshold obtained from our previous seed germination experiments, and was used to simulate the stress effect of extreme high calcium on seedlings in karst habitats); three nitrogen (N) levels: 3.75 mM (low N), 7.5 mM (normal N), and 15 mM (high N); and three nitrogen forms (nitrate-to-ammonium ratios): sole ammonium (0:100), mixed nitrate-ammonium (50:50), and sole nitrate (100:0). This resulted in a total of 18 treatment combinations. The nutrient solutions were precisely formulated using reagents including Ca(NO_3_)_2_, KNO_3_, NH_4_H_2_PO_4_, KH_2_PO_4_, NH_4_Cl, CaCl_2_, and MgSO_4_, ensuring consistent ionic strength and micronutrient concentrations across all treatments. The pH of all solutions was adjusted uniformly to 6.0. Dicyandiamide was added to the nutrient solutions to inhibit nitrification of the ammonium. Each treatment was replicated three times biologically, with each replicate consisting of 10 seedlings. The treatment period lasted for 21 days, during which the nutrient solution was replaced with a fresh supply every two days to maintain stable nutrient concentrations.

### 2.3 Measurements

#### 2.3.1 Morphological parameters

Upon completion of the treatment period, plant height (cm), root length (cm), and the leaf length (cm) of the third fully expanded compound leaf were measured. The plants were then separated into shoots and roots. Fresh weight (g) was recorded for each part. Subsequently, the samples were inactivated at 105 °C and dried at 80 °C to a constant weight to determine the dry weight (g).

#### 2.3.2 Physiological parameters

The peroxidase (POD) activity and malondialdehyde (MDA) content in leaves were determined following the methods described by Li (2020). The unit of POD activity is U/(g·min)^-1^, and the unit of MDA content is μmol/g. Superoxide dismutase (SOD) activity was measured using a commercial assay kit, the unit is U·g^-^¹. For key enzymes involved in nitrogen metabolism: nitrate reductase (NR) activity was determined using the *in vitro* method, with a unit of μg(g·h)^-1^ (Gao, 2003); glutamine synthetase (GS) activity was assayed colorimetrically, with a unit of A540/(g·h) (Zou, 2000). A portable chlorophyll meter was used to measure the SPAD value of functional leaves, serving as an indicator of relative chlorophyll content.

#### 2.3.3 Mineral element content

Dried root and leaf samples were ground and sieved. The contents of calcium and magnesium were determined using atomic absorption spectrometry according to the Determination of nitrogen, phosphorus and potassium in plants (Ministry of Agriculture of the People's Republic of China, 2011), with a unit of mg·L^-^¹. The contents of carbon and nitrogen were determined using an elemental analyzer, with a unit of mg·L^-^¹.

### 2.4 Data analysis

All data are presented as the mean ± standard deviation. A three-way analysis of variance (ANOVA) was performed using SPSS 25.0 software, and significant differences among treatments were assessed using Duncan’s multiple range test. Graphs were generated using Origin 2021 software.

## 3 Results

### 3.1 Analysis of variance for the interactive effects of calcium level, nitrogen level, and nitrogen form

The results of the three-way ANOVA (**Table 1**) revealed that the main effects of calcium level, nitrogen level, and nitrogen form exerted highly significant influences on all measured parameters (P < 0.001). Among these factors, calcium level—particularly the 120 mM high-calcium treatment—acted as a critical threshold determining the efficacy of nitrogen regulation. Under normal calcium conditions, a significant interaction was observed between nitrogen level and nitrogen form. In contrast, under high calcium stress, the regulatory effects of nitrogen were completely overridden. Significant or highly significant effects from the two-way interactions—namely Ca × N level, Ca × N form, and N level × N form—were detected across multiple aspects, including biomass, enzyme activities, photosynthetic parameters, and mineral element accumulation. Furthermore, the three-way interaction exhibited highly significant regulatory effects on root length, biomass, the activities of NR and GS, and the accumulation of Ca, Mg, N, and C.

**Table 1:** Three-way analysis of variance on the growth and physiological responses of *T.sinensis* seedlings.

Factor		Calcium level	Nitrogen level	Nitrogen form	Calcium level×Nitrogen level	Calcium level×Nitrogen form	Nitrogen level×Nitrogen form	Calcium level×Nitrogen level×Nitrogen form
df		1	2	2	2	2	4	4
F-value	Root length	115.22***	116.42***	15***	43.54***	74.92***	24.94***	52.73***
	Plant height	253.7***	9.92***	24.05***	1.39ns	22.1***	10.33***	19.56***
	Length of compound leaf	471.29***	86.67***	20.6***	63.55***	17.28***	11.33***	21.14***
	Above-ground fresh weight	686.06***	9.9***	47.46***	103.13***	5.43**	33.94***	20.17***
	Below-ground fresh weight	503.91***	1.73ns	23.58***	51.77***	8.6***	4.65**	6.91***
	Total plant fresh weight	972.38***	7.43**	37.21***	121***	6.85**	20.92***	19.86***
	Above-ground dry weight	659.55***	3.62*	69.27***	3.54*	27.18***	5.72**	2.92*
	Below-ground dry weight	582.54***	1.03ns	15.51***	6.5**	1.7ns	3.04*	2.85*
	Total plant dry weight	862.92***	2.4ns	47.31***	5.43**	15.72***	5.85***	2.22ns
	Chlorophyll SPAD value	1854.09***	5.131*	484.21***	3.389*	441.45***	11.54***	15.47***
	MDA content	1333.37***	0.3ns	0.07ns	0.49ns	0.35ns	0.06ns	0.11ns
	POD activity	1513.79***	14.99***	118.43***	10.6***	170.72***	14.02***	7.21***
	SOD activity	336.673***	6.7**	15.923***	8.759***	12.386***	0.378ns	0.994ns
	GS activity	10693.56***	66.62***	2500.55***	65.74***	2500.92***	71.1***	71.2***
	NR activity	3306.74***	225.93***	99.75***	246.33***	81.31***	58.6***	61.18***
	Root calcium content	96700.66***	1100.54***	1780.79***	775.04***	4457.74***	777.68***	369.56***
	Leaf calcium content	139469.16***	1370.19***	763.74***	5105.36***	811.42***	1620.49***	441.15***
	Root magnesium content	9031.84***	168.06***	1669.46***	152.35***	201.58***	432.29***	515.8***
	Leaf magnesium content	26335.07***	145.8***	208.26***	348.1***	571.77***	664.74***	18.35***
	Root Ca/Mg ratio	3248.79***	115.92***	498.19***	229.67***	1006.09***	416.78***	260.3***
	Leaf Ca/Mg ratio	11339.01***	341.45***	24.75***	1679.44***	113.29***	1156.11***	389.43***
	Root nitrogen content	20.05***	17.28***	9.92***	14.99***	12.52***	3.53**	6.95***
	Root carbon content	35.97***	47.6***	34.83***	7.35**	18.36***	8.45***	12.93***
	Root C/N ratio	0.071ns	1.388ns	0.15ns	10.31***	1.86ns	0.38ns	1.89ns
	Leaf nitrogen content	0.19ns	162.93***	19.26***	38.98***	2.94ns	36.18***	29.18***
	Leaf carbon content	37.708***	27.89***	25.47***	47.66***	34.56***	38.3***	6.48***
	Leaf C/N ratio	1.1ns	33.49***	4.99*	0.42ns	8.34***	9.48***	6.48***

Note: *P < 0.05 indicates a significant difference, **P < 0.01 indicates a highly significant difference, ***P < 0.001 indicates an extremely significant difference; ns indicates no significant difference (P > 0.05).

### 3.2 Effects of calcium level, nitrogen level, and nitrogen form on the morphological growth of *T.sinensis*

**Figure 1:**
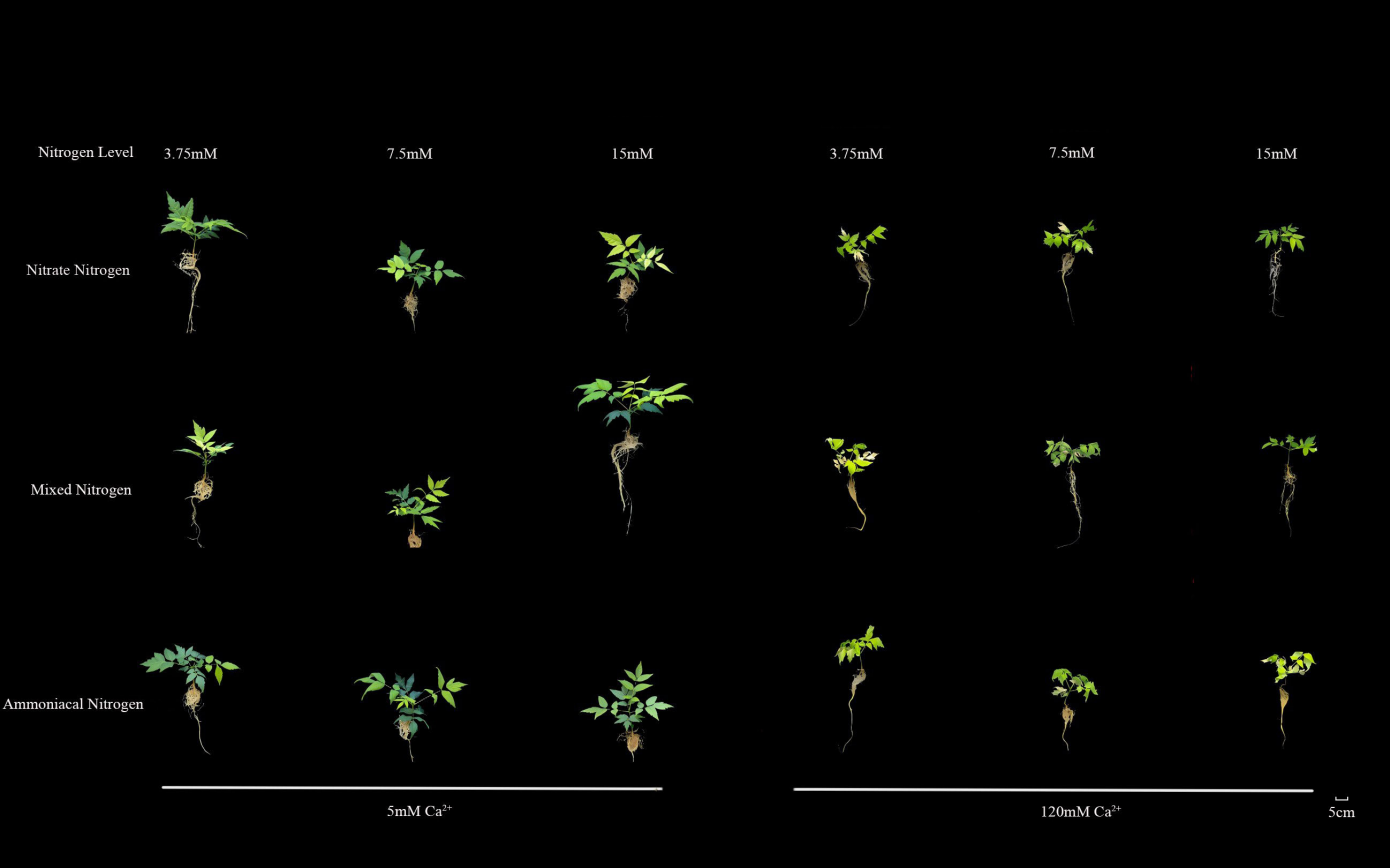
Phenotypic responses of *T.sinensis* seedlings to various treatments with different calcium levels, nitrogen levels, and nitrogen forms.

High calcium stress (120 mM) exerted an extremely strong inhibitory effect on all morphological indicators of *T.sinensis* seedlings (P<0.001) and masked the nitrogen effect. Compared with the normal calcium treatment, *T.sinensis* seedlings exhibited obvious phenotypes of growth arrest, leaf chlorosis, necrosis, and poor root development after exposure to high calcium stress (**Figure 1**), indicating that high calcium induced systemic physiological toxicity.

**Figure 2:**
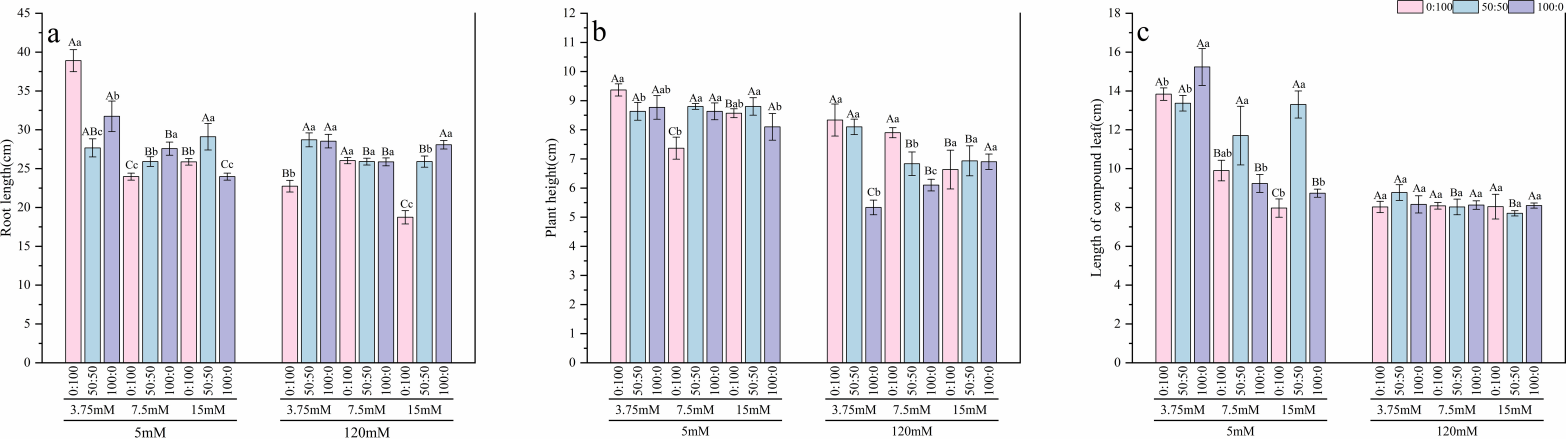
Effects of calcium level, nitrogen level, and nitrogen form on root length **(a)**, plant height **(b)**, and length of compound leaf **(c)** of *T.sinensis* seedlings. Lowercase letters indicate significant differences (p < 0.05) among different nitrogen forms within the same nitrogen level. Uppercase letters indicate significant differences (p < 0.05) among different nitrogen levels within the same nitrogen form. The same convention applies to the following figures.

**Figure 3:**
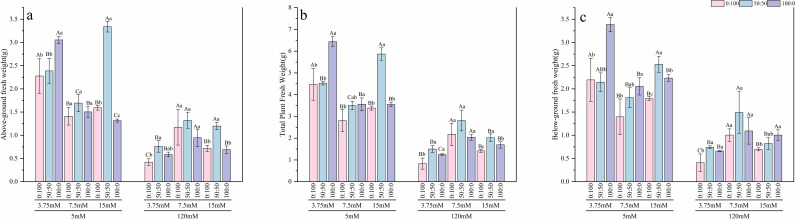
Effects of nitrogen levels and forms under different calcium levels on above-grounnd fresh weight **(a)**, below-grounnd fresh weight **(b)**, and total plant fresh weight **(c)** of *T.sinensis* seedlings.

**Figure 4:**
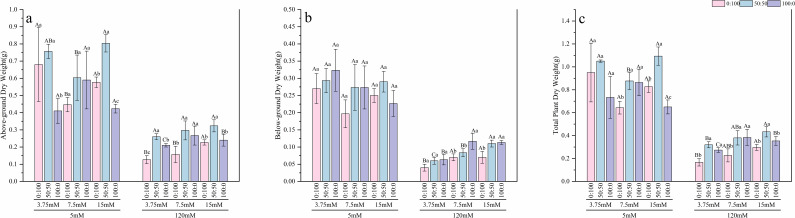
Effects of nitrogen levels and forms under different calcium levels on above-grounnd dry weight **(a)**, below-grounnd dry weight **(b)**, and total plant dry weight **(c)** of *T.sinensis* seedlings.

Under normal calcium conditions (5 mM), the regulatory effects of nitrogen were clearly observable. The response of root length (**Figure 2a**) to nitrogen form varied depending on the nitrogen level. Under low nitrogen (3.75 mM), root length was greatest with the sole ammonium treatment, showing a 40.6% increase compared to the mixed nitrogen treatment. Under normal nitrogen (7.5 mM), the sole nitrate treatment performed best, exhibiting a 13.06% increase over the sole ammonium treatment. In contrast, under high nitrogen (15 mM), the mixed nitrogen treatment resulted in the longest roots, which were 21.4% longer than those in the sole nitrate treatment. For plant height (**Figure 2b**), under low nitrogen, the sole ammonium treatment led to the maximum height, significantly outperforming the mixed nitrogen treatment by 7.89%. Regarding length of compound leaf (**Figure 2c**), under low nitrogen, the sole nitrate treatment performed optimally, showing a significant increase of approximately 9.2% compared to both the mixed and sole ammonium treatments. When the nitrogen level was raised to normal and high levels, the mixed nitrogen source demonstrated the best performance, with length of compound leaf significantly exceeding that of both sole nitrogen source treatments. In terms of biomass (**Figures 3**, **4**), results for both fresh and dry weight consistently indicated that under low nitrogen stress, sole nitrogen sources (either ammonium or nitrate) might promote growth in specific organs (roots or shoots). However, under normal and high nitrogen levels, the mixed nitrogen source most effectively promoted the coordinated growth of both above-ground and below-ground biomass, thereby maximizing the total biomass.

However, high calcium stress significantly inhibited the growth of *T.sinensis* seedlings. Average root length decreased by 10%, average plant height significantly declined by 18%, and average length of compound leaf was significantly reduced by 29% compared to the normal calcium treatment. Under high calcium stress, the average fresh weight of shoots, roots, and the whole plant decreased by approximately 60%, while the average dry weight of shoots and the whole plant declined by 63%, and root dry weight decreased by 70%. In this stressful condition, the mixed nitrogen source (50:50) treatment showed a certain mitigating effect, although biomass values remained below those observed under normal calcium levels.

In summary, high calcium treatment completely reversed the growth patterns observed under normal calcium. All morphological indices sharply declined, and the differences among treatments with varying nitrogen levels and forms were largely diminished. This indicates that high calcium stress acts as the dominant growth-limiting factor.

### 3.3 Effects of calcium level, nitrogen level, and nitrogen form on the antioxidant system and oxidative damage in *T.sinensis*

**Figure 5:**
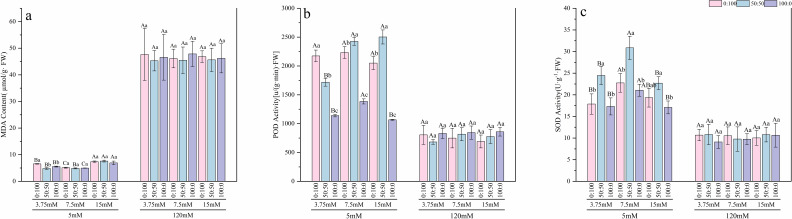
Effects of nitrogen levels and forms under different calcium levels on MDA content **(a)**, POD activity **(b)**, and SOD activity **(c)** in *T.sinensis* seedlings.

Under normal calcium conditions, the response of the antioxidant system was regulated by the nitrogen environment. At a low nitrogen level, the sole ammonium treatment induced higher POD (**Figure 5b**) activity, which was 1.91 times that of the sole nitrate treatment. However, this was accompanied by an increase in MDA content (**Figure 5a**), which was 26.73% higher than in the mixed nitrogen treatment, suggesting the induction of mild oxidative stress. Under normal and high nitrogen levels, the mixed nitrogen treatment maintained relatively high activities of SOD (**Figure 5c**) and POD (approximately 2 times and 1 time the activities of the sole nitrate treatment at the same nitrogen level, respectively), while keeping MDA levels low. This indicates that the mixed nitrogen source helped the seedlings maintain cellular redox homeostasis.

However, high calcium stress completely disrupted this balance. Compared to normal calcium levels, the average MDA content in roots under high calcium surged dramatically to 7.68 times that of the normal calcium treatment. In contrast, the activities of the key defense enzymes POD and SOD significantly decreased by 2.37-fold and 2.1-fold, respectively. No significant differences (p > 0.05) were observed among treatments for MDA content, POD activity, or SOD activity under high calcium stress. This indicates that the oxidative burst induced by high calcium exceeded the plant’s self-regulatory capacity, leading to the collapse of the antioxidant defense system.

### 3.4 Effects of calcium level, nitrogen level, and nitrogen form on nitrogen metabolism and photosynthetic efficiency in *T.sinensis*

**Figure 6:**
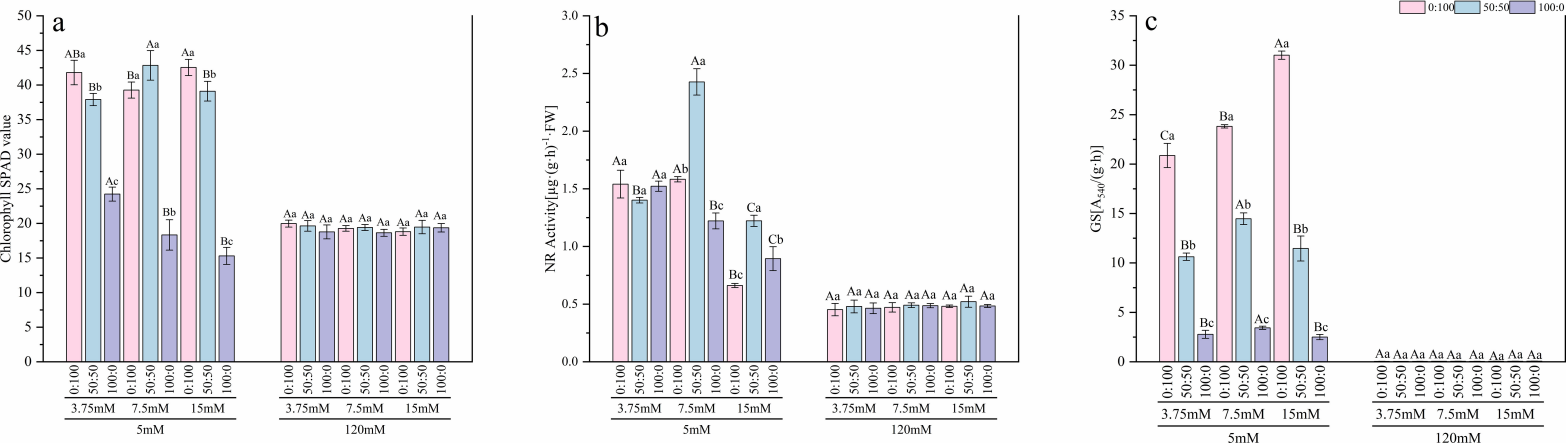
Effects of nitrogen levels and forms under different calcium levels on chlorophyll SPAD value **(a)**, NR activity **(b)**, and GS activity **(c)** in *T.sinensis* seedlings.

Under normal calcium conditions, nitrogen metabolism and photosynthesis were sensitive to nitrogen form. For seedlings supplied with the mixed nitrogen source at normal and high nitrogen levels, nitrate reductase (NR) activity (**Figure 6b**) was significantly higher than in those receiving sole nitrogen sources. The highest NR activity was observed under normal nitrogen with the mixed source, showing a 49.66% increase compared to the sole nitrate treatment at the same nitrogen level. Under sole nitrogen sources, high nitrogen stress strongly suppressed NR activity. Glutamine synthetase (GS) activity (**Figure 6c**) decreased as the proportion of nitrate increased. At a high nitrogen level, GS activity under the sole ammonium treatment was 91.95% higher than under the sole nitrate treatment. Chlorophyll SPAD values (**Figure 6a**) were higher under mixed nitrogen or sole ammonium treatments (64.03% higher than the sole nitrate treatment under high nitrogen), correlating with photosynthetic capacity.

High calcium stress inflicted devastating damage to the nitrogen metabolism and photosynthetic systems. Compared to normal calcium conditions, high calcium stress reduced the average NR activity by 65.29% and nearly completely inhibited GS activity, with a decrease of 99.66%, indicating a blockage in the primary assimilation pathway of inorganic nitrogen. Concurrently, chlorophyll SPAD values were consistently suppressed, declining by 42%, reflecting damage to the photosynthetic apparatus. At this level, high calcium once again masked any regulatory effects of nitrogen.

### 3.5 Effects of calcium level, nitrogen level, and nitrogen form on mineral element accumulation in *T.sinensis*

**Figure 7:**
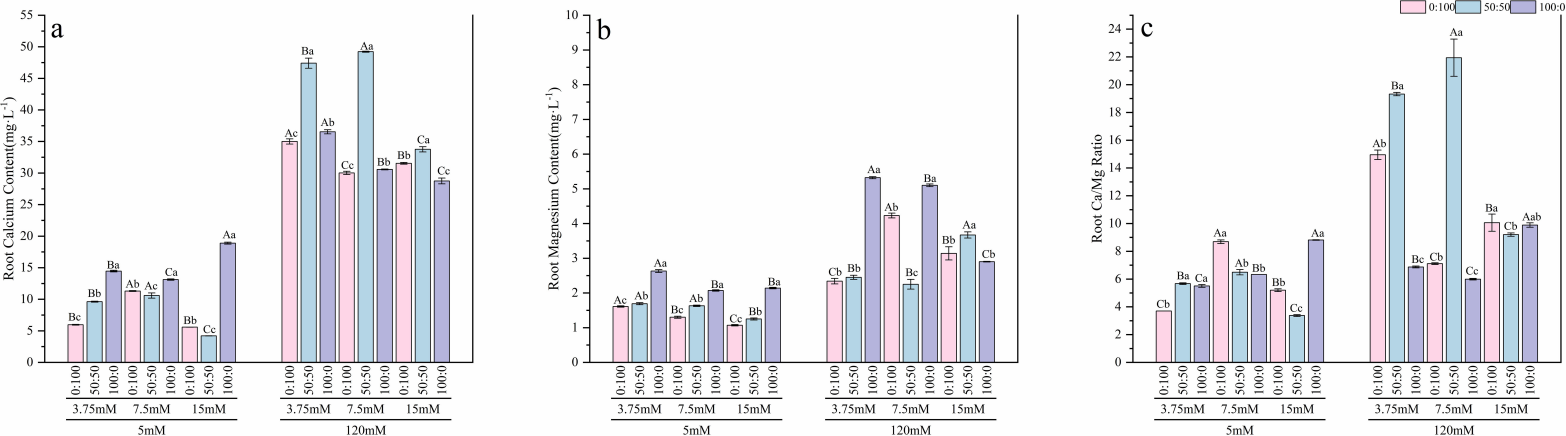
Effects of nitrogen levels and forms under different calcium levels on root calcium content **(a)**, root magnesium content **(b)**, and the root Ca/Mg ratio **(c)** in *T.sinensis* seedlings.

**Figure 8:**
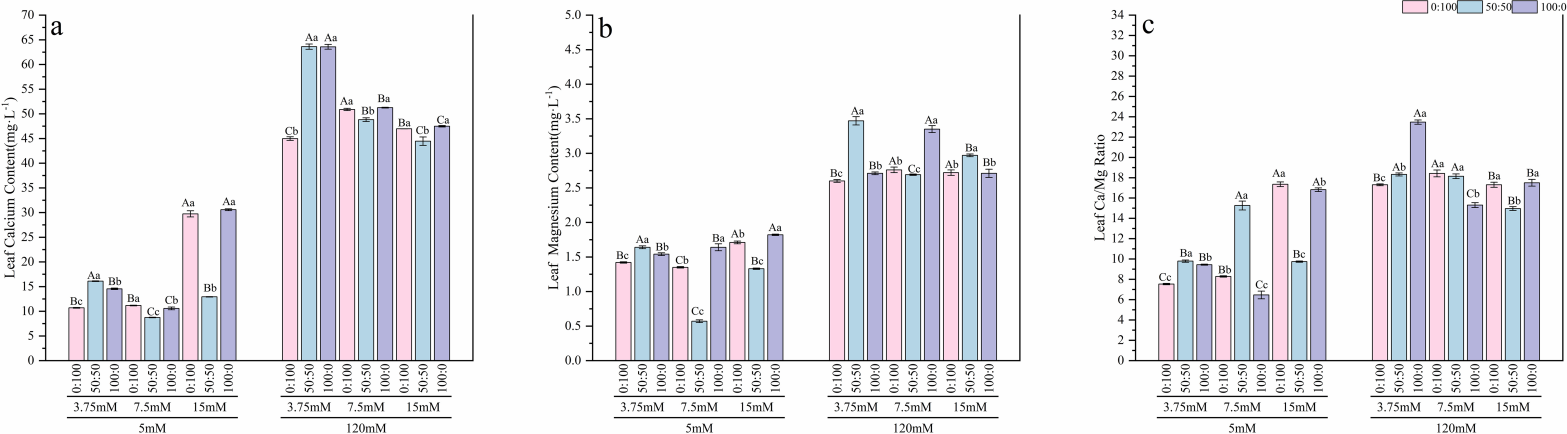
Effects of nitrogen levels and forms under different calcium levels on leaf calcium content **(a)**, leaf magnesium content **(b)**, and the leaf Ca/Mg ratio **(c)** in *T.sinensis* seedlings.

**Figure 9:**
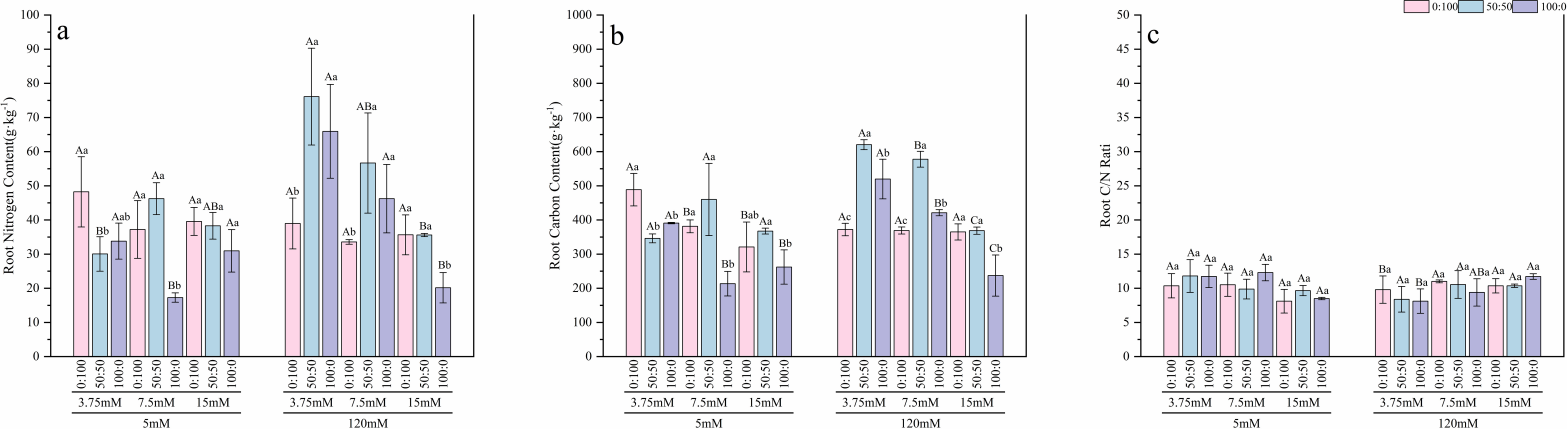
Effects of nitrogen levels and forms under different calcium levels on root nitrogen content **(a)**, root carbon content **(b)**, and the root C/N ratio **(c)** in *T.sinensis* seedlings.

**Figure 10:**
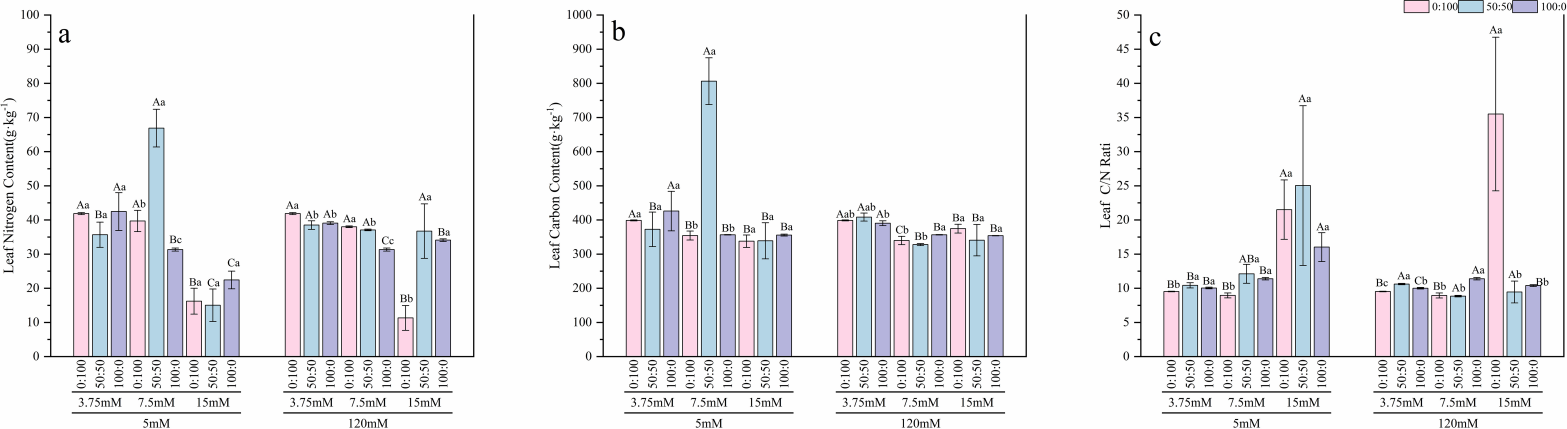
Effects of nitrogen levels and forms under different calcium levels on leaf nitrogen content **(a)**, leaf carbon content **(b)**, and the leaf C/N ratio **(c)** in *T.sinensis* seedling.

Under normal calcium conditions, nitrogen form influenced the uptake of mineral elements. The sole nitrate treatment promoted the strongest absorption of calcium and magnesium (**Figures 7**, **8**) across all nitrogen levels, with average calcium and magnesium contents being significantly higher—by factors of 2.04 and 1.72, respectively—compared to the sole ammonium treatment. Regarding the allocation of carbon and nitrogen nutrition, different nitrogen forms elicited varied responses to nitrogen concentration:

At the low nitrogen level, roots of seedlings supplied with sole ammonium exhibited the highest C and N contents (**Figures 9**, **10**), which were 1.61 and 1.41 times higher, respectively, than those in the mixed nitrogen treatment, while no significant difference (p > 0.05) was observed in the leaves. Under the normal nitrogen level, both root and leaf C and N contents reached their peak values, significantly outperforming the sole nitrate treatment. Leaf N and C contents were 2.14 and 2.26 times higher, respectively, than those in the sole nitrate treatment. At the high nitrogen level, no significant differences (p > 0.05) in C and N accumulation were found among the different nitrogen form treatments. The root C/N ratio remained stable across treatments and was unaffected by nitrogen level or form. However, the high nitrogen treatment led to a significant increase in the average leaf C/N ratio, which rose to 20.85.

High calcium stress profoundly altered the patterns and balance of element accumulation. Under high calcium stress, the average Ca and Mg contents in the roots of *T.sinensis* increased to 3.45 times and 2.04 times, respectively, of those under normal calcium conditions. Similarly, in the leaves, the contents increased to 2.64 times and 1.96 times, respectively. More critically, high calcium stress significantly disrupted the Ca/Mg balance. The average Ca/Mg ratio in roots and leaves sharply increased from 6.08 and 12.79 under normal calcium to 10.28 and 16.95, respectively, under high calcium stress, indicating a severe disruption of ion homeostasis. However, this abnormal elevation in root Mg content could be partially alleviated under specific treatment combinations: low nitrogen (3.75 mM) with either sole ammonium or mixed nitrogen, and normal nitrogen (7.5 mM) with mixed nitrogen.

Furthermore, under high calcium stress, the accumulation patterns of C and N in both roots and leaves became complex and inconsistent, indicating a breakdown in the coordination of carbon and nitrogen metabolism. Particularly under the high nitrogen with sole ammonium treatment, the leaves exhibited an extremely high C/N ratio of 35.5, suggesting severely hampered nitrogen assimilation while carbon fixation continued relatively unabated, leading to a severe metabolic imbalance.

## 4 Discussion

This study reveals the deterministic role of calcium level in shaping the responses of *T.sinensis* seedlings to nitrogen availability, delineating a response spectrum ranging from “physiological plastic adaptation” to “metabolic collapse.” Under a normal calcium background, *T.sinensis* exhibited considerable physiological plasticity, finely adjusting its nitrogen uptake and assimilation strategies to adapt to varying nitrogen supply conditions. However, when the calcium level increased to a critical threshold, it triggered a cascading collapse spanning from membrane system damage to a paralysis of core metabolic functions, thereby completely overriding any regulatory effects of nitrogen form. This dichotomous response pattern profoundly reflects the adaptation limits and hierarchical mechanisms of plants facing combined stress.

### 4.1 Adaptive strategies under normal calcium conditions: nitrogen level-dependent physiological plasticity

Under normal calcium conditions, the growth of *T.sinensis* seedlings was significantly regulated by nitrogen level and form, exhibiting a clear “nitrogen level-dependent” pattern. The plants dynamically adjusted their growth and resource allocation strategies to adapt to varying nitrogen environments, reflecting a strong ecological plasticity in *T.sinensis*.

Nitrogen deficiency activated a “root survival strategy,” prioritizing resource allocation to root system expansion. Results showed that under low nitrogen, root length increased by 21.2% and 19.7% compared to normal and high nitrogen levels, respectively, reaching its maximum under the sole ammonium treatment. This phenomenon can be attributed to the low energy cost associated with the direct assimilation of ammonium, coupled with the maintenance of relatively high GS activity in the seedlings, enabling efficient assimilation of ammonium. Concurrently, higher POD activity and chlorophyll SPAD values helped maintain oxidative balance and photosynthetic potential, promoting the accumulation of C and N nutrients in the roots. This provided a sufficient material and energetic foundation for root construction (Schneider et al., 2021). However, this preferential allocation of resources to the roots directly suppressed morphological growth of the shoots (seedling height, biomass). This illustrates the classical trade-off mechanism where plants dynamically adjust their source-sink relationships under extreme nutrient limitation to ensure survival (Bloom et al., 1985; Brouwer, 1962).

Under normal and high nitrogen levels, plants shift toward a “balanced growth” strategy. At these nitrogen levels, *T.sinensis* seedlings supplied with mixed nitrate-ammonium nutrition (50:50) demonstrated optimal performance in both growth morphology and physiological metabolism. This phenotypic advantage stems from the synergistic effects of multiple physiological mechanisms. First, the balanced uptake of the anion (NO_3_^-^) and cation (NH_4_^+^) helps maintain intracellular ionic equilibrium and pH homeostasis. The complementary nature of the mixed nitrogen source effectively mitigates ionic imbalance induced by high nitrate, promotes the absorption and transport of NH_4_^+^, and thereby alleviates the disruption of cellular ion homeostasis caused by ammonium toxicity (Rivero-Marcos, 2025; Zhu et al., 2021; Xiao et al., 2022). Second, the concurrent operation of the nitrate and ammonium assimilation pathways can alleviate feedback inhibition inherent to a single pathway, optimizing nitrogen metabolic flux. This metabolic optimization not only enhances nitrogen use efficiency but also sustains high activities of NR and antioxidant enzymes, establishing a robust antioxidant defense system (Feng et al., 2020; Rivero-Marcos et al., 2026). Finally, the mixed nitrogen source is more conducive to coordinating carbon and nitrogen metabolism, providing sufficient metabolic substrates for antioxidant and other defense systems, and thereby promoting seedling height and biomass accumulation (Zhang et al., 2022; Wei et al., 2024).

### 4.2 Collapse mechanism under high calcium: cascading physiological dysregulation

High calcium stress exerted inhibitory effects on *T.sinensis* seedlings that exceeded their tolerance threshold, leading to significant reductions in plant height, root length, and biomass. The overall appearance of the plants was characterized by growth arrest and wilting. This macroscopic phenotype is the integrated outcome of the sequential disruption and eventual collapse of multiple internal physiological and metabolic networks, revealing the adaptive limits of *T.sinensis* to this calcium concentration.

Firstly, membrane damage and oxidative burst synergistically exacerbated the wilting symptoms. High calcium stress directly compromised the integrity of cell membranes, leading to the leakage of electrolytes and metabolites, manifested as reduced water uptake capacity and severe wilting of the shoots. This was accompanied by a burst in reactive oxygen species (ROS) production. This study found that root MDA content surged to 7.68 times the level under normal conditions. This dramatic increase in MDA, a hallmark product of lipid peroxidation, confirms severe physical damage to the membrane system. The collapse of membrane structure not only signifies the loss of selective permeability but also disrupts cellular compartmentalization. This leads to the massive release of excess Ca²^+^, originally sequestered in vacuoles or cell walls, into the cytosol, triggering subsequent cascading damage (Demidchik, 2018; Farmer and Mueller, 2013).

Secondly, disruption of calcium signaling and collapse of the antioxidant defense system occurred. The sharp rise in cytosolic Ca²^+^ levels under high calcium stress fundamentally destabilizes its role as a second messenger, transforming it from a key regulatory signal into a cellular toxic agent. Confronted with severe oxidative stress and this signaling disorder, the plant attempts to activate its antioxidant enzyme systems, such as SOD and POD. However, contrary to the typical defensive response, the activities of these enzymes under high calcium were significantly lower than those under normal conditions. This decline in enzymatic activity directly impairs the plant’s capacity to scavenge ROS, which in turn further accelerates the accumulation of MDA, establishing a detrimental vicious cycle. This inactivation of the primary defense system likely stems from excessive cytosolic Ca²^+^ interfering with the gene expression of antioxidant enzymes or disrupting the active site conformation of the enzyme proteins themselves (Bonomelli et al., 2021), ultimately depriving the seedlings of their fundamental ability to neutralize ROS.

Thirdly, the blockage of core metabolic pathways leads to the interruption of material and energy supply. Current research indicates that disrupted calcium signaling, by affecting the activity of calmodulin and various calcium-dependent or calcium-regulated protein kinases (such as CDPKs and MAPKs), directly or indirectly alters the phosphorylation status of nitrate reductase (NR) and glutamine synthetase (GS) (Campos et al., 2022; Liu et al., 2022). This study found that under severe high-calcium stress, this imbalance in phosphorylation status leads to NR and GS activities being suppressed to extremely low levels. Inhibition of NR activity means the nitrate reduction pathway is blocked, while the loss of GS activity directly severs the assimilation channel for ammonium, causing a complete paralysis of the conversion from inorganic to organic nitrogen. Concurrently, severe ion imbalance, particularly the extremely high Ca/Mg ratio, damages chloroplast structure and inhibits the activity of key chlorophyll synthesis enzymes (such as magnesium chelatase), leading to a loss of photosynthetic capacity (Li et al., 2025; Parab et al., 2023; Qi et al., 2025).

This “dual paralysis” of nitrogen assimilation and carbon fixation fundamentally severs the material and energy sources required for plant growth, leaving the plant unable to repair damaged cells and accelerating the progression toward death. This directly explains the observed phenotypes of significant reductions in seedling height, root length, compound leaf length, and biomass. The apparent contradiction between growth arrest and carbon/nitrogen accumulation reveals a metabolic “pseudo-homeostasis” under extreme stress. Although the plants exhibited wilting and chlorosis, their carbon and nitrogen contents and ratios did not differ significantly from those under normal calcium conditions. Combined with the aforementioned biomass stagnation, this does not indicate normal metabolism. We speculate that this apparent phenomenon of metabolic homeostasis may arise from the combined effects of the following two aspects. On one hand, the severely inhibited growth rate leads to a halt in carbon and nitrogen consumption (Sugiura et al., 2024). On the other hand, residual photosynthesis and delayed protein degradation (mediated by Hsp protective mechanisms) cause passive accumulation of carbon and nitrogen in source organs (El Omari, 2022; Zhong et al., 2024). This phenomenon, where “arrested growth leads to reduced consumption,” masks the underlying reality of “disrupted metabolic flux,” similar to the increase in non-structural carbohydrates (NSC) observed under drought stress rather than a decrease (Tsuji et al., 2021).However, this study only determined the static C/N ratio and did not trace the dynamics of carbon and nitrogen metabolic flux, changes in growth rate, or isotopic fractionation characteristics. Therefore, the hypothesis of “growth stagnation–reduced consumption–passive accumulation” cannot be verified. Accordingly, this explanation remains a scientific speculation to be validated and requires verification using techniques such as ¹³C/¹^5^N isotopic tracing and dynamic sampling.

Finally, the metabolic collapse-dominant effect and the loss of physiological basis for nitrogen regulation. The synergistic effects of the above processes resulted in the complete collapse of ion homeostasis, redox homeostasis, and metabolic homeostasis in *T.sinensis* seedlings, pushing the plants into a state of physiological failure. Under this condition, high calcium disrupted the core metabolic network, including membrane system damage, ROS burst, inactivation of nitrogen metabolism enzymes, and destruction of photosynthetic apparatus. Consequently, the growth promotion and metabolic regulation targeted by optimizing nitrogen forms lost their physiological basis.

### 4.3 Implications for agricultural practice

Based on the results of this study, we suggest that *T.sinensis* cultivation in karst regions should be managed based on the level of soil exchangeable calcium. For normal soils, a NO_3_^-^:NH_4_^+^ ratio of 50:50 should be preferentially adopted. However, in high-calcium soils, plants exhibit limestone-induced stress responses, and simply adjusting nitrogen forms has limited alleviating effects. Priority should be given to soil improvement measures (e.g., applying organic manure, using sulfur powder to adjust soil pH and reduce available calcium content, thereby mitigating high-calcium stress) or selecting calcium-tolerant cultivars. Nitrogen management can be further optimized after soil calcium activity is reduced.

## 5 Conclusions

This study clearly demonstrates that the response of *T.sinensis* seedlings to combined calcium and nitrogen stress exhibits a concentration-dependent shift in the dominant limiting factor. Under normal calcium conditions (5 mM), nitrogen level and form act as key plastic regulators, with a mixed nitrate-to-ammonium ratio (50:50) being optimal at medium and high nitrogen levels (7.5–15 mM). Under high calcium stress (120 mM), calcium becomes the dominant limiting factor, triggering a cascade of metabolic collapse and rendering nitrogen regulation ineffective. As these findings are derived from a hydroponic seedling experiment, field validation should fully consider the influences of natural environmental factors, including soil microbial community structure, soil water dynamics, and soil physicochemical properties. Based on these results, the following agricultural practice recommendations are proposed: For soils with normal calcium content, a 1:1 nitrate-to-ammonium ratio is recommended for precision fertilization to optimize the growth of *T.sinensis* seedlings. For habitats under high calcium stress, priority should be given to soil improvement measures (e.g., application of organic manure and calcium passivators) or breeding of calcium-tolerant *T.sinensis* cultivars, rather than simply alleviating stress by adjusting nitrogen forms.

## Data Availability

The datasets Mendeley for this study can be found in the Interactive Effects of Calcium-Nitrogen Combined Stress and Nitrogen Forms on Nitrogen Metabolism and Physiological Ecological Response Mechanisms of *Toona sinensis* Seedlings (https://data.mendeley.com/preview/wbzpks38nv?a=8a141f2a-18c5-4efb-86a0-830908df06df).
