# Supplementary material for: Interactive effects of calcium-nitrogen combined stress and nitrogen forms on nitrogen metabolism and physiological ecological response mechanisms of Toona sinensis Seedlings
Source: Front Plant Sci. 2026 Apr 10;17:1806019. doi: 10.3389/fpls.2026.1806019 (PMC13107976; doi:10.3389/fpls.2026.1806019)
Supplement: Supplementary file 1 [file Table1.docx]

Appendix A

Appendix A.1

**Table A1.** Three-way Analysis of Variance on the Growth and Physiological Responses of *T.sinensis* Seedlings.

| **Factor** | | **Calcium level** | **Nitrogen level** | **Nitrogen form** | **Calcium level×Nitrogen level** | **Calcium level×Nitrogen form** | **Nitrogen level×Nitrogen form** | **Calcium level×Nitrogen level×Nitrogen form** |
| --- | --- | --- | --- | --- | --- | --- | --- | --- |
| **df** | | 1 | 2 | 2 | 2 | 2 | 4 | 4 |
| **F-value** | **Root length** | 115.22*** | 116.42*** | 15*** | 43.54*** | 74.92*** | 24.94*** | 52.73*** |
|  | **Plant height** | 253.7*** | 9.92*** | 24.05*** | 1.39ns | 22.1*** | 10.33*** | 19.56*** |
|  | **Length of compound leaf** | 471.29*** | 86.67*** | 20.6*** | 63.55*** | 17.28*** | 11.33*** | 21.14*** |
|  | **Above-grounnd fresh weight** | 686.06*** | 9.9*** | 47.46*** | 103.13*** | 5.43** | 33.94*** | 20.17*** |
|  | **Below-grounnd fresh weight** | 503.91*** | 1.73ns | 23.58*** | 51.77*** | 8.6*** | 4.65** | 6.91*** |
|  | **Total plant fresh weight** | 972.38*** | 7.43** | 37.21*** | 121*** | 6.85** | 20.92*** | 19.86*** |
|  | **Above-grounnd dry weight** | 659.55*** | 3.62* | 69.27*** | 3.54* | 27.18*** | 5.72** | 2.92* |
|  | **Below-grounnd dry weight** | 582.54*** | 1.03ns | 15.51*** | 6.5** | 1.7ns | 3.04* | 2.85* |
|  | **Total plant dry weight** | 862.92*** | 2.4ns | 47.31*** | 5.43** | 15.72*** | 5.85*** | 2.22ns |
|  | **Chlorophyll SPAD value** | 1854.09*** | 5.131* | 484.21*** | 3.389* | 441.45*** | 11.54*** | 15.47*** |
|  | **MDA content** | 1333.37*** | 0.3ns | 0.07ns | 0.49ns | 0.35ns | 0.06ns | 0.11ns |
|  | **POD activity** | 1513.79*** | 14.99*** | 118.43*** | 10.6*** | 170.72*** | 14.02*** | 7.21*** |
|  | **SOD activity** | 336.673*** | 6.7** | 15.923*** | 8.759*** | 12.386*** | 0.378ns | 0.994ns |
|  | **GS activity** | 10693.56*** | 66.62*** | 2500.55*** | 65.74*** | 2500.92*** | 71.1*** | 71.2*** |
|  | **NR activity** | 3306.74*** | 225.93*** | 99.75*** | 246.33*** | 81.31*** | 58.6*** | 61.18*** |
|  | **Root calcium content** | 96700.66*** | 1100.54*** | 1780.79*** | 775.04*** | 4457.74*** | 777.68*** | 369.56*** |
|  | **Leaf calcium content** | 139469.16*** | 1370.19*** | 763.74*** | 5105.36*** | 811.42*** | 1620.49*** | 441.15*** |
|  | **Root magnesium content** | 9031.84*** | 168.06*** | 1669.46*** | 152.35*** | 201.58*** | 432.29*** | 515.8*** |
|  | **Leaf magnesium content** | 26335.07*** | 145.8*** | 208.26*** | 348.1*** | 571.77*** | 664.74*** | 18.35*** |
|  | **Root Ca/Mg ratio** | 3248.79*** | 115.92*** | 498.19*** | 229.67*** | 1006.09*** | 416.78*** | 260.3*** |
|  | **Leaf Ca/Mg ratio** | 11339.01*** | 341.45*** | 24.75*** | 1679.44*** | 113.29*** | 1156.11*** | 389.43*** |
|  | **Root nitrogen content** | 20.05*** | 17.28*** | 9.92*** | 14.99*** | 12.52*** | 3.53** | 6.95*** |
|  | **Root carbon content** | 35.97*** | 47.6*** | 34.83*** | 7.35** | 18.36*** | 8.45*** | 12.93*** |
|  | **Root C/N ratio** | 0.071ns | 1.388ns | 0.15ns | 10.31*** | 1.86ns | 0.38ns | 1.89ns |
|  | **Leaf nitrogen content** | 0.19ns | 162.93*** | 19.26*** | 38.98*** | 2.94ns | 36.18*** | 29.18*** |
|  | **Leaf carbon content** | 37.708*** | 27.89*** | 25.47*** | 47.66*** | 34.56*** | 38.3*** | 6.48*** |
|  | **Leaf C/N ratio** | 1.1ns | 33.49*** | 4.99* | 0.42ns | 8.34*** | 9.48*** | 6.48*** |
